# Enhanced Diclofenac Biodegradation by Bacterial Strains and a Microbial Consortium from Activated Sludge: Toxicity Assessment and Insights into Microbial Community Dynamics

**DOI:** 10.3390/jox16010024

**Published:** 2026-02-02

**Authors:** Alba Lara-Moreno, Belen Rodriguez-Morillo, Fernando Madrid, Pedro M. Martin-Sanchez, Jaime Villaverde, Carmen Mejías, Esteban Alonso, Juan Luis Santos, Esmeralda Morillo

**Affiliations:** 1 Department of Microbiology and Parasitology, Faculty of Pharmacy, University of Seville, 41012 Seville, Spain; 2 Institute of Natural Resources and Agrobiology of Seville, Department of Agrochemistry, Environmental Microbiology and Soil Conservation, Spanish National Research Council (IRNAS-CSIC), 41012 Seville, Spain; belenrodriguezmorillo@gmail.com (B.R.-M.); fmadrid@irnase.csic.es (F.M.); pmartin@irnase.csic.es (P.M.M.-S.); jvillaverde@irnase.csic.es (J.V.); 3 Department of Analytical Chemistry, University of Seville, C/Virgen de África 7, 41011 Seville, Spain; cmpadilla@us.es (C.M.); ealonso@us.es (E.A.); jlsantos@us.es (J.L.S.)

**Keywords:** diclofenac, bioremediation, activated sludge, DCF-degrading bacteria, DCF-degrading consortium, ecotoxicity, microbial community

## Abstract

Diclofenac (DCF) is a widely used non-steroidal anti-inflammatory drug whose presence in environmental matrices has led to its classification as an emerging contaminant. Developing effective and sustainable removal strategies is therefore essential. In this study, *Pseudomonas aeruginosa* CSWD.1, *Pseudomonas* sp. CSWD.2, and a microbial consortium (MC) were isolated from activated sludge through enrichment cultures with DCF and employed as laboratory models to investigate DCF biodegradation capacity under a biosafety-aware framework. Biodegradation assays supplemented with glucose showed limited removal (45.5%) by CSWD.1, whereas CSWD.2 and the MC achieved complete elimination (100%) of 10 mg L^−1^ DCF in 21 and 5 days, respectively. Three extracellular metabolites, 4’-hydroxy-diclofenac (4’-OH-DCF), 5-hydroxy-diclofenac (5-OH-DCF), and putative NO_2_-DCF, were detected, with concentrations varying during degradation. Persistence of 4’-OH-DCF and tentatively identified NO_2_-DCF after 28 days was potentially associated with increased toxicity relative to the abiotic control. Overall, the results suggest that evaluating metabolites and their toxicity is essential, requiring isolation of additional microorganisms able to degrade 4’-OH-DCF and NO_2_-DCF to combine with the microorganisms isolated in this study. Metabarcoding analysis of the microbial consortium after bioremediation revealed the dominant bacterial population of *Burkholderia* (88.9% relative abundance) and a predominant fungal genus *Talaromyces* (80.1%), indicating that both bacteria and fungi may be associated with DCF transformation. These results provide insights into microbial community dynamics and their potential application in designing effective consortia for DCF bioremediation.

## 1. Introduction

Pain is one of the prevalent symptoms treated pharmacologically and represents a major global public health concern. In Europe, approximately 27% of the adult population suffers from chronic pain [1]. Among the therapeutic options available, anti-inflammatory drugs have been used since ancient times, with non-steroidal anti-inflammatory drugs (NSAIDs) remaining essential for managing both acute and chronic inflammation and pain. More than 50 NSAIDs are currently marketed worldwide, with ibuprofen, naproxen, aspirin, and DCF being among the most widely consumed [2]. DCF is the arylacetic acid derivative 2-[2-(2,6-dichloroanilino)phenyl]acetic acid (C_14_H_11_Cl_2_NO_2_). Its pharmacological activity is based on the inhibition of prostaglandin synthesis through the time-dependent suppression of cyclooxygenase enzymes (COX-1 and COX-2), with a preferential suppression of COX-2 over COX-1, thereby reducing the conversion of arachidonic acid into pro-inflammatory mediators [3]. Due to its effectiveness and accessibility, DCF is frequently used for self-medication. In a study by Benites-Meza et al. [4], 68.21% of 2158 participants reported taking NSAIDs without medical supervision, with DCF being one of the most common choices. Global consumption of DCF is estimated at 1443 ± 58 tons per year, with Europe accounting for approximately 27.8% [5]. The widespread and often unregulated use of pharmaceuticals, combined with insufficient medical oversight, has contributed to their increasing detection in environmental matrices, leading to the classification of many of these compounds, including DCF, as emerging environmental contaminants [6,7].

DCF enters the environment primarily through effluents from hospitals, pharmaceutical industries, and wastewater treatment plants (WWTPs) that receive human and animal waste [8,9]. Reported concentrations in wastewater range from ng L^−1^ to μg L^−1^ [10,11]. Numerous studies have demonstrated that DCF is a recalcitrant compound, exhibiting low removal efficiencies in conventional biological treatment systems [12,13]. Consequently, DCF and its metabolites persist in surface waters, groundwater, and even drinking water in various regions worldwide [14,15,16]. Furthermore, its presence in biosolids and treated wastewater reused for agricultural purposes raises additional concerns regarding soil contamination [17,18].

The environmental recalcitrance of DCF is largely attributed to its chemical structure, as supported by the findings of Kimura et al. [19], who reported that the removal efficiency of active pharmaceutical compounds strongly depends on molecular features such as the number of aromatic rings and the presence of chlorine substituents. DCF contains two aromatic rings and two chlorine atoms, which confer high molecular stability. Aromatic systems are inherently resistant to enzymatic cleavage, while carbon–chlorine bonds are powerful and poorly reactive toward microbial enzymes. In addition, the presence of electron-withdrawing groups further reduces the susceptibility of DCF to oxidative and hydrolytic processes, thereby limiting its biodegradation under environmental and engineered conditions. As a result, conventional biological treatment systems often fail to remove DCF efficiently [1,19,20].

Beyond its intrinsic chemical stability, several physicochemical and operational factors contribute to the persistence of DCF in WWTPs. DCF exhibits relatively high hydrophobicity (logK_ow_ ≈ 4.5), which can promote sorption onto sludge solids [20]. Additionally, previous studies have shown that microbial activity and hydrolytic processes during wastewater treatment may transform DCF-conjugated metabolites, leading to the regeneration of the parent compound. This phenomenon contributes to the apparent persistence of DCF and, in some cases, to observed increases in its concentration along the treatment train [21,22].

Given its persistence and widespread occurrence, the development of effective and sustainable decontamination strategies is increasingly important. Biodegradation, defined as “a process that relies on biological mechanisms to reduce (degrade, detoxify, mineralize, or transform) pollutant concentrations to an innocuous state” [23], has gained attention as a promising approach. Bioaugmentation (microbial inoculation) and biostimulation (addition of macronutrients and micronutrients), in particular, offer advantages such as high efficiency, environmental compatibility, and cost-effectiveness [24,25]. However, despite the extensive environmental presence of DCF, relatively few bacterial strains capable of degrading it have been identified. This limitation is largely attributed to the compound’s adverse effects on microbial physiology, including membrane disruption, enzymatic inhibition, and oxidative stress caused by reactive oxygen species (ROS), which damage lipids, proteins, and nucleic acids and trigger cellular stress responses [18,26,27]. Nevertheless, some bacterial genera, including *Pseudomonas* [26,28], *Rhodococcus* [29], *Labrys* [30], *Raoultella* [31], *Stenotrophomonas* [32], and *Klebsiella* [33], have demonstrated the ability to transform DCF through pathways such as hydroxylation, dehydroxylation, decarboxylation, and dichlorination, producing metabolites such as 4′-OH-DCF, 5-OH-DCF, 3′-hydroxy-diclofenac, 4′,5-dihydroxydiclofenac, and quinone imine [18,34]. Reported degradation efficiencies vary widely: *Pseudomonas* sp. DCα4 showed highly efficient biotransformation of DCF, achieving up to 99.8% removal within 68 h from an initial concentration of 10 mg L^−1^. Nevertheless, complete mineralization was not confirmed. This process was primarily driven by N–C bond hydrolases rather than classical oxidative enzymes such as cytochrome P450s, laccases, or peroxidases. Genomic analyses of *Pseudomonas* sp. DCα4 have identified several genes encoding hydrolases (*puuD, atzF, astB, nit1, nylB*), corresponding to γ-glutamyl-GABA hydrolase (EC 3.5.1.94), allophanate hydrolase (EC 3.5.1.54), N-succinyl arginine dihydrolase (EC 3.5.3.23), deaminated glutathione amidase (EC 3.5.1.128), and 6-aminohexanoate-dimer hydrolase (EC 3.5.1.46). These enzymes are amidohydrolases that hydrolyze C–N bonds in linear compounds and, in the case of N-succinyl arginine dihydrolase, in cyclic/imidazolidine derivatives. Given that diclofenac contains an aromatic amide bond, these hydrolases could potentially contribute to its transformation, although their activity against DCF has not yet been experimentally confirmed [28]. *Rhodococcus ruber* IEGM 346 degraded approximately 25 mg L^−1^ of DCF over 60 days from an initial concentration of 50 mg L^−1^. The results were supported by measurements of *Rhodococcus* respiratory activity, indicating cell viability and metabolic intensity, confirming that DCF decomposition was entirely dependent on the catalytic activity of the cells and was catalyzed by oxygenases [29]. In a study with *Raoultella* sp. DD4, approximately 0.6 mg L^−1^ of DCF was transformed in 28 days. However, no information was provided regarding metabolite formation or the DCF degradation pathway [31]. In another study, *Labrys portucalensis* F11 biotransformed approximately 24 µM of DCF over 30 days. Based on the identified intermediates, a metabolic pathway was proposed. DCF degradation was primarily initiated through hydroxylation reactions, producing hydroxy-DCF isomers mediated by monooxygenases and dioxygenases, followed by dehalogenation, methylation, decarboxylation, and oxidation, generating a variety of intermediates. Importantly, the metabolite profile indicated stoichiometric liberation of chlorine, suggesting complete degradation of the pharmaceutical compound [30]. Similarly, *Klebsiella* sp. KSC degraded 70 mg L^−1^ of DCF in 72 h. The main degradation pathways involved hydroxylation via cytochrome P450, decarboxylation, and partial dechlorination of the central ring of DCF [33]. Co-metabolic conditions, such as the presence of readily available carbon sources, have also been shown to enhance pharmaceutical biodegradation by promoting microbial growth and metabolic activity [35]. As observed for DCF, *Stenotrophomonas humi*, exposed to 1.5 mg L^−1^ of DCF in the presence of 3 g L^−1^ glucose, achieved 75.1% degradation of DCF within 8 days, forming a transformation product corresponding to nitro-DCF [32], and *Pseudomonas moorei* KB4 removed 0.5 mg L^−1^ of DCF in 12 days, with an increase in DCF removal to 1 mg L^−1^ was achieved after 11 days in the presence of glucose. The transformation was mediated by a suite of oxygenases, including monooxygenases, dioxygenases, and cytochrome P450 enzymes; however, complete mineralization was not demonstrated in this study [26].

In some cases, microbial consortia outperform single strains by completely degrading contaminants, enhancing their metabolic versatility and overall efficiency in the concurrent removal of diverse compounds. This has also been described for DCF [36]. Microbial consortia have achieved high removal efficiencies (80–98%) across a wide range of initial DCF concentrations, from 10 to 150 mg L^−1^. Biodegradation processes were mainly associated with hydroxylation reactions, generating metabolites with low ecotoxicity, and also exhibiting tolerance to variable environmental conditions, such as pH and temperature. These findings highlight the potential of microbial consortia as a robust and efficient strategy for DCF removal in biological treatment systems [36,37,38]. In this context, advances in high-throughput amplicon sequencing, using marker genes such as 16S rRNA for bacteria and Internal Transcribed Spacer (ITS) for fungi, have enabled high-resolution characterization of the taxonomic composition of microbial consortia involved in the degradation of pharmaceuticals. This approach overcomes the known limitations of culture-dependent methods, providing a more comprehensive view of the diversity and dynamics of microbial communities participating in biodegradation processes [25,39,40]. The integration of DNA metabarcoding with chemical analyses is essential for monitoring shifts in microbial community composition, identifying key degraders, and assessing the remediation potential of the applied consortia [41,42].

Due to the extensive environmental dispersion of DCF, its environmental fate and ecotoxicological evaluation should be considered a priority [11,18]. Moreover, as reported by Badawy et al. [18], certain transformation products formed during DCF degradation have been reported to exhibit higher toxicity than the original compound, thereby potentially intensifying environmental contamination.

Activated sludge, collected from aeration tanks, was selected as a rich and diverse microbial reservoir since WWTPs are often exposed to pharmaceutical compounds [43], imposing selective pressure on the bacterial community and potentially selecting microorganisms with the ability to survive in the presence of these compounds. Based on this, the objectives of the study were as follows: (i) to establish enrichment cultures from activated sludge using DCF as the selective substrate, to obtain a microbial consortium and bacterial isolates capable of DCF degradation. Selection criteria included sustained growth in the presence of DCF and measurable reduction in DCF concentration; (ii) to evaluate DCF degradation efficiency in aqueous solutions and to identify the metabolites produced during the biodegradation process, including transformation pathways such as hydroxylation, glucuronidation, lactam formation, nitration, nitrosation, and subsequent aromatic ring modifications; (iii) to assess the ecotoxicological impact of DCF before and after biodegradation, using *Aliivibrio fischeri* as a test organism, thereby evaluating the effectiveness of the decontamination strategy in terms of toxicity mitigation; and (iv) to investigate microbial community dynamics during DCF degradation using 16S rRNA and ITS amplicon sequencing, to identify the key bacterial and fungal taxa potentially involved in DCF biotransformation and to link microbial composition with observed degradation patterns.

This study offers interdisciplinary insights by integrating microbiology, analytical chemistry, toxicology, and metabarcoding to advance the understanding of DCF biodegradation, a contaminant that remains insufficiently characterized in terms of its microbial degraders, transformation pathways, and associated toxicity.

## 2. Materials and Methods

### 2.1. Material

An analytical standard of DCF sodium salt (C_14_H_10_Cl_2_NNaO_2_, purity > 98%, CAS: 15307-79-6) was obtained from Sigma-Aldrich (Madrid, Spain). Luria–Bertani (LB) broth and mineral salt medium (MSM) were prepared using analytical-grade reagents (Sigma-Aldrich, Madrid, Spain) and sterilized by autoclaving at 120 °C, inlet pressure of 103 kPa for 20 min before use (steam autoclave Auster-G, P-Selecta ST DRY PV-III). The LB broth formulation (per liter of deionized water) consisted of 10 g tryptone, 5 g yeast extract, and 10 g NaCl (BD Difco™, Fisher Scientific, Pittsburgh, PA, USA). MSM was composed of the following components (per liter of deionized water): 0.5 g of KH_2_PO_4_, 0.5 g of K_2_HPO_4_, 0.01 g of NaCl, 0.2 g of MgCl_2_·6 H_2_O, 0.02 g of CaCl_2_, 1 g of (NH_4_)_2_SO_4_, 0.339 mg of MnSO_4_, 0.428 mg of ZnSO_4_, 0.347 mg of (NH_4_)_6_Mo_7_O_24_·4 H_2_O, 0.4 mg of CoCl_2_·6 H_2_O, 5 mg of FeSO_4_ and 7 H_2_O, and 0.2 mg of CuSO_4_·5 H_2_O. The pH was adjusted to 7.0 ± 0.2 with NaOH.

For this study, activated sludge was collected from the San Jerónimo WWTP (Seville), operated by the company EMASESA (Seville, Spain). This plant processes wastewater from the northern area of Seville. It receives effluents from sources such as the Virgen Macarena University Hospital in Seville and from some municipalities of Seville province.

### 2.2. Methods

#### 2.2.1. Enrichment and Isolation of DCF-Degrading Consortium and DCF-Degrading Strains

A microbial consortium, along with individual strains capable of degrading DCF, was obtained through enrichment cultures using activated sludge from the San Jerónimo WWTP. The enrichment culture procedure was carried out following the methodology described by Bessa et al. [44], with some modifications. Briefly, sterile 250 mL Erlenmeyer flasks (autoclave Auster-G, P-Selecta with one cycle at 120 °C, inlet pressure of 103 kPa, for 20 min) were inoculated with 10 mL of activated sludge and 150 mL of MSM, supplemented with DCF at an initial concentration of 24 mg L^−1^. DCF concentration used to enrich cultures exceeds the concentrations typically observed in WWTPs but was selected to create a selective pressure on the bacterial community, facilitating the enrichment and isolation of strains capable of tolerating this compound [40]. As a control, an additional treatment was prepared without the addition of sludge. The cultures were incubated with orbital shaking (150 rpm) at 30 °C. Aliquots of supernatant (1 mL) were collected on days 7, 14, and 21 to quantify the remaining DCF concentration by HPLC (see Section 2.2.5). Additionally, aliquots (0.2 mL) from each flask (every triplicate separately) were taken on days 14 and 21. These aliquots were pooled into a single sample, and a 0.2 mL sample of this mixture was used for the isolation of bacterial degraders. After 21 days, the culture was divided into three subcultures: one was used to continue monitoring DCF under the same conditions, while the other two were supplemented with 3 g L^−1^ of additional carbon sources (glucose or yeast extract). Although this concentration is higher than would typically be found in the environment, it could be safely applied in a real treatment scenario if needed. After 7 and 14 days of incubation (28 and 35 days after the start of the experiment), a 1 mL aliquot was used to monitor the DCF concentration as described above, and microbial isolation was performed. A degrading MC and 4 isolates were obtained after 7 days of enrichment in the presence of DCF and glucose.

To obtain the microbial consortium and to isolate bacterial strains, MSM agar plates were employed. Once the agar had solidified, 0.5 mL of an MSM solution containing 200 mg L^−1^ DCF, as the only added source of carbon and energy, was spread evenly on each plate using a sterile spreader until fully absorbed. Subsequently, 0.2 mL of culture from the different enrichments was streaked on the described plates and incubated at 30 °C for 3 days. Successive colony isolations were performed on LB plates incubated at 30 °C under aerobic conditions, recognizing and selecting different morphotypes according to their size, color, edge, and elevation of colonies. Once purified, the selected isolated strains and microbial consortium were preserved in cryovials (Microbank™ 2D) at −80 °C until further use.

In parallel, a second enrichment assay in a 250 mL Erlenmeyer flask with 50 mL of MSM was conducted with a higher initial DCF concentration (200 mg L^−1^) to increase the selective pressure on the microbial community [45]. The culture was incubated with orbital shaking (150 rpm) at 30 °C, and every 15 days, 5 mL of the culture was transferred to a new flask containing 45 mL of MSM supplemented with 200 mg L^−1^ DCF and incubated again. On days 15 and 30, a 0.2 mL aliquot was taken for bacterial isolation as previously described. Isolated bacteria were labeled with codes based on the research group (CSW: CONSOWAT), the contaminant used for isolation (Diclofenac: D), and a sequential isolation number.

#### 2.2.2. Molecular Identification of Bacterial Isolates

To identify DCF-degrading bacteria, total genomic DNA was isolated from 1 mL of pure LB cultures using the G-spin™ Total DNA Extraction Kit (iNtRON Biotechnology, Inc., Kirkland, WA, USA). The bacterial strains were identified by sequencing the 16S rRNA gene (~1465 bp), which was amplified via PCR with universal primers 27F (5′-AGAGTTTGATCCTGGCTCAG-3′) and 1492R (5′-GGTTACCTTGTTACGACTT-3′) [46]. Amplified PCR products were sequenced through the Sanger method at STABVIDA laboratory (Caparica, Portugal), using the primers 518F (5′-CCAGCAGCCGCGGTAATACG-3′) and 800R (5′-TACCAGGGTATCTAATCC-3′). The resulting 16S rRNA sequences were deposited in the NCBI GenBank database and used for molecular identification by BLASTn (BLAST+ 2.17.0) search on the NCBI website.

#### 2.2.3. Inhibitory Concentration of DCF for Bacterial Growth

The half-maximal inhibitory concentration (IC_50_) for bacterial growth represents the amount of DCF required to inhibit growth by 50% [47]. To determine this value, experiments were carried out in duplicate using sterile 50 mL Erlenmeyer flasks, each containing 10 mL of LB medium supplemented with different concentrations of DCF sodium salt (10, 100, 500, 1000, 3000, and 5000 mg L^−1^) to cover a wide range, from low levels to high levels considered potentially toxic. Cultures were inoculated with bacteria or a consortium adjusted to an initial optical density at 600 nm (OD_600_) of 0.1. A parallel control without DCF was included to represent normal growth conditions.

All cultures were incubated at 30 °C for 24 h under aerobic conditions with agitation at 180 rpm. After incubation, bacterial growth was quantified by measuring OD_600_ with a VWR UV-3100 spectrophotometer (VWR International, Radnor, PA, USA). Cell viability (%) was expressed as the ratio between the OD_600_ of each treated culture and the OD_600_ of the control culture. The IC_50_ values were calculated from the dose–response curves generated by plotting microbial viability (%) against the logarithm of diclofenac concentration (mg L^−1^). Each dataset was fitted with a second-order polynomial regression as follows:

y = a(log[DCF])^2^ + b(log[DCF]) + c

where y represents microbial viability (%), log[DCF] is the logarithm of the DCF concentration (mg L^−1^), and a, b, and c are the experimentally determined regression coefficients. To determine IC_50_, a value of y = 50 was substituted into the equation, the corresponding log[DCF] value was calculated, and the antilogarithm was applied to obtain the DCF concentration associated with 50% growth inhibition.

#### 2.2.4. DCF Biodegradation Test in Solution

Biodegradation assays were conducted in sterile 250 mL Erlenmeyer flasks covered with a cotton plug containing 50 mL of MSM solution supplemented with glucose (3 g L^−1^), spiked with 10 mg L^−1^ DCF sodium salt, and inoculated with the microbial culture at a volume sufficient to achieve an initial OD_600_ of ~1.0, measured using a VWR UV-3100 spectrophotometer. In parallel, an abiotic control was established under identical conditions but without inoculation. A total of 12 flasks were used, distributed as follows: (i) isolate CSWD.1, (ii) isolate CSWD.2, (iii) microbial consortium (MC), and (iv) abiotic control, all in triplicate. Treatments were incubated at 30 °C with orbital shaking at 180 rpm.

For the preparation of microbial culture, isolated bacterial strains and the microbial consortium, previously stored in cryovials at −80 °C, were inoculated into 250 mL flasks containing 50 mL of LB medium with DCF sodium salt (10 mg L^−1^) and incubated at 30 °C and 180 rpm under aerobic conditions for 24 h. After incubation, the cultures were centrifuged at 7000 rpm for 15 min. The supernatant was discarded, and the bacterial pellet was washed twice with MSM to completely remove residual DCF, possible metabolites, and residual LB medium. An additional centrifugation step was performed under the same conditions. Finally, the pellet was resuspended in 1 mL of MSM. To monitor residual DCF concentration and the formation of metabolites, 1 mL aliquots of the supernatant were collected at the initial time and at 0.5, 1, 2, 5, 7, 14, 21, and 28 days, and they were quantified as described in detail in Section 2.2.5. At the end of the biodegradation assay (28 days), the remaining solutions from the flasks were collected in 15 mL Falcon tubes and centrifuged. Supernatants were stored at −20 °C for subsequent ecotoxicity assays.

The biodegradation curves were evaluated and fitted to the most suitable kinetic models using the tool developed by the FOCUS [48] working group for degradation kinetics. Curve fitting was performed with the GRG nonlinear algorithm in the Solver add-in, and model parameters were optimized through the least-squares method. The observed degradation profiles were best described by two first-order kinetic models: the Simple First-Order (SFO) model and the biphasic First-Order model, commonly referred to as the Hockey-Stick (HS) model. These models are expressed as follows:

M_t_ = M_0_·e^−kt^ (SFO)

DT_50_ = ln 2/k (SFO)

M_t_ = M_0_ e^−k1^ tb e^−k2^(t − tb) (HS)

DT_50_ = (ln 100/100 − 50)/k_1_     if DT_50_ ≤ tb (HS)

DT_50_ = tb + (ln 100/100 − 50) − k_1_ tb)/k_2_     if DT_50_ > tb (HS)

In these equations, M_t_ represents the DCF concentration (mg L^−1^) at time t, and M_0_ is the initial concentration immediately after spiking. The degradation rate constants are denoted by k (for SFO) or k_1_ and k_2_ (for the HS model), corresponding to the fast and slower degradation phases, respectively. The parameter tb indicates the breakpoint at which the degradation rate shifts. The DT_50_, or half-life, represents the time required for the DCF concentration to decrease by 50% relative to its initial level.

#### 2.2.5. Analytical Methods

The remaining DCF concentration was quantified by HPLC, as described by Dorado et al. [49]. The supernatant was transferred to 2 mL Eppendorf tubes and centrifuged at 12,000 rpm for 2 min. DCF quantification was performed using HPLC (Shimadzu LC-2010 HT, Shimadzu Corporation, Kyoto, Japan) equipped with a 150 mm × 4 mm octadecylsilane (ODS) column with 5 μm particle size and a programmable UV detector set at 285 nm. Analyses were conducted at room temperature using a mobile phase composed of acetonitrile–water (65:35) with 0.8 mL L^−1^ orthophosphoric acid. A 20 μL injection volume was used for each sample. External linear calibration was performed, and the limit of quantification was 0.05 mg L^−1^.

The analysis of DCF metabolites was performed by liquid chromatography tandem mass spectrometry (LC-MS/MS). The instrument was an Agilent 1200 HPLC system with a vacuum degasser, a binary pump, an autosampler, and a thermostated column compartment (Agilent, Santa Clara, CA, USA). Mass spectrometry analysis was carried out on an Agilent 6410 triple-quadrupole mass (QqQ) spectrometer equipped with an electrospray ionization (ESI) source. The separation of compounds was carried out by the injection of 10 µL of sample in a Kinetex Polar C18 analytical column (50 mm × 3.0 mm i.d., 2.6 µm particle size) (Phenomenex, Torrance, CA, USA), thermostated at 30 °C and protected by a Polar C18 guard column (SecurityGuard ULTRA Cartridges 2 mm × 3 mm i.d.) (Phenomenex, Torrance, CA, USA). Chromatographic analysis was carried out using gradient elution at a flow rate of 0.3 mL min^−1^ using a mobile phase composed of 10 mM ammonium acetate (solvent A) and acetonitrile (solvent B). Elution started with 15% of solvent B, held for 1 min; then increased to 55% in 5 min, held for 2 min; then, to 97% in 1 min and held for 2 min. Return to initial conditions was performed in 1 min and held for 5 min for re-equilibration. Total run time was 17 min.

Target compounds were DCF, 4’-OH-DCF, and 5-OH-DCF. For the ionization of the compounds, the following settings were applied: MS capillary voltage: 3500 V in positive and 2500 V in negative, drying-gas flow rate: 9 L min^−1^, drying-gas temperature: 350 °C, nebulizer pressure: 35 psi and cell accelerator voltage: 4 V. Fragmentor was set at 60 V. Each extract was measured using the Multiple Reaction Monitoring (MRM) mode. Two transitions were selected for each target compound. MRM1 was used for quantification, and MRM2 and the ratio were used for confirmation. Conditions applied in MRM mode are shown in Table S1. Although 4’-OH-DCF and 5-OH-DCF exhibit the same transitions, chromatographic separation allowed them to be quantified. For proper identification of the compounds, standard solutions of each compound were injected individually. Calibration curves were constructed by the injection of at least seven calibration standards. For quality control, blanks and spiked samples were injected throughout the analysis. The limit of quantification was 0.5 µg L^−1^ for DCF and 0.1 µg L^−1^ for 4’-OH-DCF and 5-OH-DCF. The identification of additional DCF metabolites/transformation products (TPs), whose presence could be inferred from previous studies but for which analytical standards were not available, was conducted according to Osorio et al. [50]. The metabolites analyzed were as follows: 1-O-acylglucuronide diclofenac, 4,5-dihydroxydiclofenac, TP339 (nitration), TP323 (nitrosation), and 5-hydroxydiclofenac lactam. Each extract was analyzed in MRM mode, with specific conditions detailed by Osorio et al. [50] (Table S2), and the results for these compounds are reported in a semi-quantitative manner using peak area only. Structures of DCF, metabolites, and TPs have been reported in Table S3.

#### 2.2.6. Ecotoxicity Bioassays

The inhibitory effect of DCF and/or potential metabolites produced during the biodegradation process was assessed using a bioluminescence-based assay with the bacterium *Aliivibrio fischeri*, following the standard ISO 11348–3 basic test protocol [51]. For that purpose, supernatant samples were collected at the beginning (abiotic control, consisting of MSM spiked with 10 mg L^−1^ DCF and 3 g L^−1^ of glucose) and at the end of the 28-day biodegradation experiment (treatments with *P. aeruginosa* CSWD.1, *Pseudomonas* sp. CSWD.2, and the microbial consortium MC). All samples exhibited an initial pH of 7.0 ± 0.2, which was not modified during incubation. The solutions were filtered to remove particles and serially diluted (1:2) with 2% NaCl to final concentrations of 90.9%, 45.45%, 22.73%, and 11.36% (*v*/*v*). Changes in bioluminescence were measured using a MICROTOX Model 500 analyzer (Modern Water Inc., New Castle, DE, USA) after 15 min of contact between the samples and *A. fischeri*, and compared to a control without DCF. EC_50_ values (the concentration of DCF (% *v*/*v*) causing toxic effects on 50% of the bacterial population) were calculated and expressed in Toxic Units (TU) = 100/EC_50_. The TU results were classified according to the categories proposed by Persoone et al. [52].

#### 2.2.7. Characterization of Microbial Consortium by DNA Metabarcoding

DNA was extracted from microbial pellet samples collected at the beginning (“before”) and the end (“after” 28 days) of the DCF biodegradation experiment using the DNeasy PowerSoil Pro Kit (Qiagen GmbH, Hilden, Germany). DNA concentrations of the extracts were quantified using the fluorometric Qubit dsDNA BR assay (Invitrogen—Life Technologies, Eugene, OR, USA) according to the manufacturer’s instructions and subsequently diluted to achieve concentrations of around 5–10 ng µL^−1^. The selected barcodes, V3 and V4 regions of the 16S marker for bacteria, and the ITS2 region for fungi, were amplified using primers that included Illumina adapter overhang sequences attached to the target-specific sequences. Bacteria-specific sequences Bact341F (5′-CCTACGGGNGGCWGCAG-3′) and Bac785R (5′-GACTACHVGGGTATCTAATCC-3′) were previously described by Klindworth et al. [53], while fungi-specific sequences ITS3-KYO2-F (5′-GATGAAGAACGYAGYRAA-3′) and ITS4-KYO1-R (5′-TCCTCCGCTTWTTGWTWTGC-3′) were designed by Toju et al. [54]. After PCR amplification, the multiplexing step was performed using the Nextera XT DNA Library Preparation Kit (Illumina, San Diego, CA, USA). After size verification of libraries (~550 bp for bacteria and ~430 bp for fungi) on a Bioanalyzer DNA 1000 chip (Agilent Technologies, Santa Clara, CA, USA), they were sequenced using a 2 × 300 bp paired-end MiSeq sequencing run using the Reagent kit v3 according to the manufacturer’s instructions (Illumina). The resulting raw sequencing data are available at the European Nucleotide Archive (ENA), EMBL-EBI, under accession no. PRJEB98466 (https://www.ebi.ac.uk/ena/browser/view/PRJEB98466, accessed on 1 January 2026). Bioinformatic analyses were conducted using the QIIME2 pipeline [55]. Quality filtering, denoising, merging of contigs, removal of chimeras, and creation of the amplicon sequence variants (ASV) table were performed using the DADA2 pipeline [56], starting from paired-end data. Taxonomic assignments of ASVs were performed using the Naive Bayesian classifier integrated in Qiime2 plugins against the bacterial database Silva138 [57] and the fungal database UNITE (v. 10 UNITE QIIME release for fungi) [58].

#### 2.2.8. Statistical Analysis

Standard deviations for biodegradation data comparison were calculated using Microsoft Excel. One-way ANOVA with three replicates was performed to study the significant differences among treatments. Statistical analysis was conducted by means of the SPSS v. 21 (SPSS Inc., Chicago, IL, USA) statistical package.

Statistical analyses of DNA metabarcoding data were conducted in R v 4.3.0 through RStudio v 2023.03.0 [59]. Tidyverse v 1.2.1 [60] and vegan v 2.6-4 [61] R packages were used for data manipulation, plotting, and ecological analyses, respectively. The quality-filtered ASV matrices were rarefied to 95,729 and 3231 reads per sample for the bacterial and fungal datasets, respectively, using the vegan function rrarefy (Table S4). The low number of fungal ITS2 sequences achieved for the sample collected before the biodegradation experiment likely reflected the low fungal biomass present in this sample. Alpha-diversity indices, such as species richness (observed number of ASVs), Shannon, and Inverse Simpson, were calculated using the functions specnumber and diversity from vegan. The overlap of the ASVs detected in the two samples (at the beginning and at the end of the biodegradation test) was illustrated using the VennDiagram R package [62]. Taxonomic composition was analyzed at phylum, order, and genus levels, avoiding the species level due to the well-known limitations of species identification based on such short DNA fragments.

## 3. Results and Discussion

### 3.1. Enrichment and Isolation of the DCF-Degrading Microbial Consortium and Bacterial Strains

Enrichment cultures were initiated using DCF as the sole added source of carbon and energy, with initial DCF concentrations of 24 mg L^−1^ and 200 mg L^−1^. The use of enrichment cultures at both low and high concentrations has been previously explored by several authors, who argue that low concentrations allow for the selection of oligotrophic bacteria and subpopulations specialized in degrading contaminants at reduced levels, typically associated with faster mineralization and shorter lag phases [63]. Conversely, high concentrations during enrichment favor the selection of bacteria that are tolerant to contaminants and can efficiently degrade them in environments with elevated pollutant loads [64].

From the enrichment performed at a high DCF concentration (200 mg L^−1^), specific potential DCF-degrading strains forming isolated colonies were selected from the MSM agar plates containing 200 mg L^−1^ DCF as the only added source of carbon and energy. Five bacteria were isolated after 15 days; after 30 days and following a medium refresh, a total of four additional colonies were formed and isolated from the plates inoculated with the enrichment cultures.

**Figure 1:**
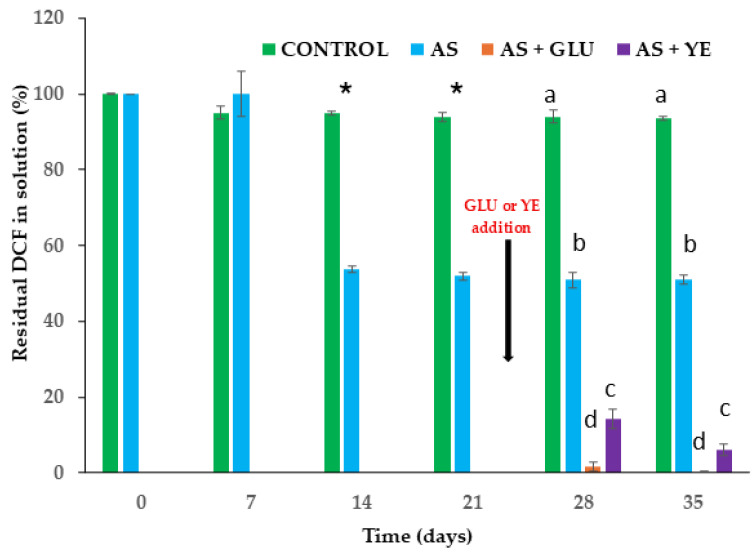
Evolution of the residual percentage of DCF (24 mg L^−1^ initial concentration) during enrichment culture, without activated sludge (control) and with activated sludge in the absence of external carbon sources (AS). After 21 days, AS was split into three groups: (i) AS, (ii) in the presence of 3 g L^−1^ glucose (AS + GLU), and (iii) in the presence of 3 g L^−1^ yeast extract (AS + YE). Error bars indicate standard deviation from the replicates. The *t*-test was applied for statistical differences at days 0, 7, 14, and 21. * indicates significant differences among control and AS treatments (*p* < 0.01). ANOVA was used for days 28 and 35. Different letters for each bar mean significant differences among the corresponding treatments (Tukey test, significance level 99%).

In cultures supplemented with 24 mg L^−1^ DCF, nearly half of the initial content was removed after 14 days (Figure 1), at which point bacterial isolation was attempted. Five different bacterial isolates were obtained. After 21 days, degradation levels were similar to those observed on day 14 (Figure 1), indicating a plateau in DCF removal. This stagnation might be attributed to a combination of substrate-related toxicity and nutrient limitation, but more research is needed to support this statement. DCF and several of its transformation products are known to induce oxidative stress and membrane damage in microorganisms, reducing metabolic activity and biodegradation capacity [26,27,65]. In addition, the accumulation of reactive or persistent transformation products, such as p-benzoquinone imines, hydroxylated DCF derivatives, or DCF-benzoic acid, may further inhibit microbial activity or limit complete mineralization [66,67,68]. After 21 days, the enrichment culture was divided into three equal parts; one of them was used in the same conditions, and the other two were supplemented with additional carbon sources: glucose or yeast extract (3 g L^−1^). In the glucose-supplemented culture, nearly 100% of DCF was degraded within 7 days after its addition, while in the yeast extract-supplemented culture, about 85% and 94% degradation was achieved after 7 and 14 days after yeast addition, respectively. These results indicate that DCF degradation was strongly enhanced under cometabolic conditions, in agreement with previous studies [24,26,27,32]. After 7 days of enrichment in the presence of DCF and glucose, four more different bacterial isolates were obtained. Based on these results, glucose was selected as the most suitable co-substrate for subsequent experiments.

In general, the presence of glucose enhances the biodegradation of DCF by serving as a source of carbon and nutrients, thereby stimulating the growth and metabolic activity of the microorganisms responsible for its degradation [35]. Several studies have demonstrated that the addition of glucose as a co-metabolite significantly increases the rate of DCF removal by bacteria, thereby improving the efficiency of drug elimination compared to single-substrate conditions. For instance, in the presence of *Enterobacter hormaechei* D15, the DCF removal rate increased from 52.8% to approximately 82% upon supplementation with glucose (0.05 g L^−1^) as an external carbon source [24]. Similarly, Żur et al. [26] reported that *Pseudomonas moorei* KB4 degraded 0.51 mg L^−1^ of DCF (51%) in 12 days under standard conditions, whereas under co-metabolic conditions with glucose supplementation (1.5 g L^−1^), complete degradation of 1 mg L^−1^ (100%) was achieved within 11 days. Żur et al. [27] reported that when cultures were exposed to 50 µg L^−1^ of DCF under co-metabolic conditions with glucose at a final concentration of 1.5 g L^−1^, the percentage of DCF degradation increased markedly from 31%, 27%, and 55% in the absence of glucose to 100% in its presence for *Klebsiella huaxiensis* KB7, *Klebsiella aerogenes* KB18, and *Serratia proteamaculans* KB19, respectively. In another study, *Stenotrophomonas humi* DIC_5 removed 75.1% of DCF (1.5 mg L^−1^) within 8 days when glucose (3 g L^−1^) was used as a co-substrate, since removal in the absence of glucose was not achieved [32,69]. Nonetheless, in some cases, biodegradation was not significantly improved compared to single-substrate conditions. Żur et al. [27] investigated the enhancement of biodegradation in several bacterial isolates and found that the positive effect of co-metabolism was strain-specific. For example, when *K. huaxiensis* KB7 and *K. aerogenes* KB18 were exposed to 1.5 mg L^−1^ of DCF in the presence of glucose, the extent of biodegradation did not increase. This may occur because glucose, as an easily assimilable carbon source, can divert the bacteria from using the drug as a carbon source and instead lead them to preferentially metabolize glucose [24,70].

In our study, a microbial consortium, labeled “MC”, was obtained from the 24 mg L^−1^ DCF enrichment culture supplemented with glucose for 7 days. An aliquot was plated on MSM-agar plates supplemented with DCF to obtain a microbial consortium. In parallel, four different bacterial strains were identified in MSM-agar plates with DCF and isolated on LB-agar plates. Out of the 18 bacterial isolates obtained from all the enrichment cultures (5 after 15 days and 4 after 30 days of enrichment at a DCF concentration of 200 mg L^−1^, 5 after 14 days of enrichment at a DCF concentration of 24 mg L^−1^, and 4 after 7 days of enrichment in the presence of DCF and glucose), only two demonstrated the ability to degrade DCF (see Section 3.3). They were designated as strains CSWD.1, isolated after 30 days of enrichment at a DCF concentration of 200 mg L^−1^, and CSWD.2, isolated after 7 days of enrichment with 24 mg L^−1^ in the presence of DCF and glucose. These DCF-degrading bacterial strains were successfully identified according to the NCBI BLASTn analyses of their 16S rRNA gene sequences. CSWD.1 showed 100% identity with *Pseudomonas aeruginosa* sequences. Although *P. aeruginosa* was isolated from an environmental sample and is not expected to be cytotoxic or pathogenic under normal conditions, it is an opportunistic pathogen; therefore, its use in environmental bioaugmentation would require strict biosafety measures. In this study, it was employed as a laboratory model to investigate DCF biodegradation capacity. Similarly, the 16S rRNA gene sequence of strain CSWD.2 exhibited 99.92% identity with *Pseudomonas* sp. from the NCBI GenBank “nt” sequence database (Table 1). It is noteworthy that although the CSWD.2 sequence showed a high level of sequence identity (99.68%) with *Pseudomonas syringae* sequences, species-level identification cannot be conclusively established based solely on the 16S rRNA marker. The bacterial strains CSWD.1 and CSWD.2, along with the consortium “MC” were selected to continue with subsequent studies.

### 3.2. Diclofenac-Induced Inhibition of Bacterial Growth

**Figure 2:**
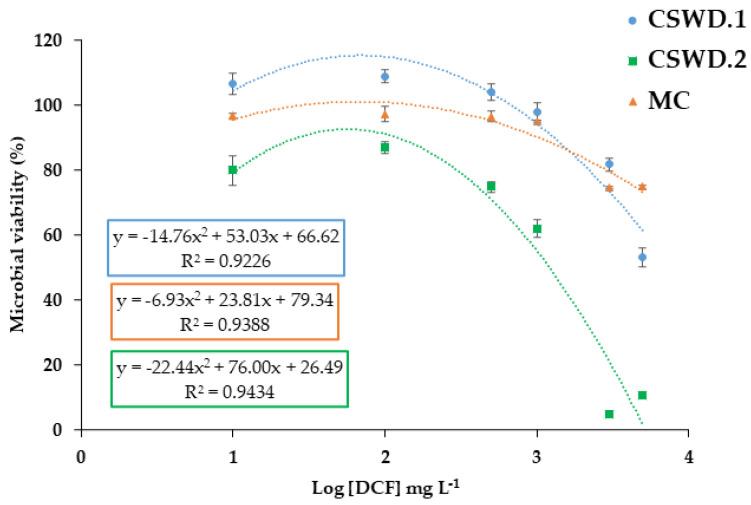
Graphical representation of the best-fitting polynomial curves for microbial viability as a function of the logarithm of DCF concentration (log [DCF]) for the two bacterial isolates and consortium. Error bars indicate standard deviation from three replicates.

DCF is well known for its anti-inflammatory properties; however, several studies have also reported that it may display antibacterial effects [27,29]. This potential activity has been supported by evidence showing its capacity to inhibit microbial growth, as demonstrated by reported IC_50_ values and growth reduction assays across different microorganisms [71,72,73]. In this work, the IC_50_ was calculated to evaluate how increasing concentrations of DCF (10, 100, 500, 1000, 3000, and 5000 mg L^−1^) influenced the bacterial strains and consortium tested (*P. aeruginosa* CSWD.1, *Pseudomonas* sp. CSWD.2, and MC, respectively). The experimental design compared OD_600_ after 24 h of incubation in the presence of DCF. The 24 h incubation period was selected based on the growth kinetics of the bacterial strains, as growth curve analyses (Supplementary Material, Figure S1) showed that strains CSWD.1 and CSWD.2 reached the stationary phase after approximately 15 h, ensuring stabilized growth before endpoint determination (24 h). Figure 2 shows bacterial viability (%) as a function of the logarithm of DCF concentration. Polynomial regression curves were obtained for each strain, as these equations provided a good fit to the experimental data, as indicated in Table 2, where the R^2^ value was greater than 0.92 in all cases. This type of nonlinear fitting has previously been reported in studies addressing multidrug resistance [74]. Overall, the results indicate a high level of DCF tolerance among the isolates. The highest IC_50_ value was observed for CSWD.1, followed by CSWD.2, with values of 7600 and 1100 mg L^−1^, respectively (Table 2). These values are considerably higher than the environmental concentrations reported in surface and wastewater, which typically range from ng L^−1^ to µg L^−1^ [18,75]. The results reveal that the CSWD.1 strain shows 100% resistance to DCF at concentrations up to approximately 500 mg L^−1^ of the drug (Figure 2). This resilience of *P. aeruginosa* was previously described by Klockgether et al. [76], who reported that this bacterium possesses enhanced genome-encoding capacity and that its metabolic versatility enables it to adapt to environmental changes and to utilize a wide range of nutrients. Conversely, CSWD.2 exhibits greater sensitivity to DCF when compared to CSWD.1. However, these results suggest that this strain can tolerate high concentrations of DCF (more than 80% resistance at 100 mg L^−1^ DCF) and may, therefore, have strong potential for its biodegradation.

Several authors have investigated the resistance of different bacterial species to DCF, reporting variable results depending on the species. According to published data, *Escherichia coli* C600 and *Staphylococcus aureus* 6571 exhibited tolerance to DCF in the range of 50–100 mg L^−1^ [73]; *Enterococcus faecalis*, approximately 50 mg L^−1^ [67]; *Mycobacterium* spp., between 10 and 25 mg L^−1^ [72]; and several *Rhodococcus* species showed a microbial inhibition concentration above 200 mg L^−1^ [29], among others. These values, however, are lower than those detected in our study, as our strains can tolerate concentrations of up to 100 mg L^−1^. Consistent with our findings, Żur et al. [27] reported that species such as *Enterobacter cloacae* KB1, *Klebsiella huaxiensis* KB7, *Klebsiella aerogenes* KB18, and *Serratia proteamaculans* KB19 exhibited high tolerance to DCF, with IC_50_ values exceeding 5 g L^−1^, even higher than that observed in our work for *Pseudomonas* sp. CSWD.2, but similar to the IC_50_ value obtained for *P. aeruginosa* CSWD.1 (Table 2).

To our knowledge, only a recent study has reported a value of minimal inhibitory concentration (MIC) of 5120 mg L^−1^ for *P. aeruginosa* clinical strains exposed to DCF [77], which is a high value in line with our result (7600 mg L^−1^) and further supports the high resistance of this species to DCF; as far as we know, no IC_50_ data for DCF has been reported for *Pseudomonas* species.

In the case of the consortium (MC), the IC_50_ value was considerably higher, reaching 25,518 mg L^−1^ (Table 2). The high IC_50_ obtained for MC reflects the overall low sensitivity of the microbial consortium to DCF, compared with the effects observed in the isolated strains. Although the IC_50_ values for individual strains were also relatively high (ranging from 7600 to 1100 mg L^−1^), they were still lower than that of the consortium (25,518 mg L^−1^). This result may be attributed to the DCF biodegradation capacity of bacterial consortia, particularly those enriched with degrading strains, where microorganisms act synergistically, potentially mitigating toxicity and reducing the ecotoxicological burden of the drug in contaminated environments [37,78]. Only one study has investigated the resistance of microbial consortia to DCF. Symsaris et al. [79] examined the sensitivity of two microbial communities, one derived from a mesophilic wastewater treatment plant sludge-based inoculum and the other from a thermophilic manure-based inoculum, through metagenomic analyses. The reported IC_50_ values were 546 mg L^−1^ and 481 mg L^−1^ for the mesophilic and thermophilic consortia, respectively. However, the consortium analyzed in the present study exhibited a markedly higher IC_50_ (25,518 mg L^−1^) than that previously reported. This substantial difference suggests that the studied consortium displays a significantly greater tolerance to DCF, possibly related to its specific microbial composition derived from the enrichment process using DCF from which it was generated, the presence of DCF-degrading strains, and the nature of its predominant metabolic processes, which metabolic pathways primarily govern the consortium’s activity and tolerance toward DCF [78,80].

### 3.3. Diclofenac Biodegradation in Solution

**Figure 3:**
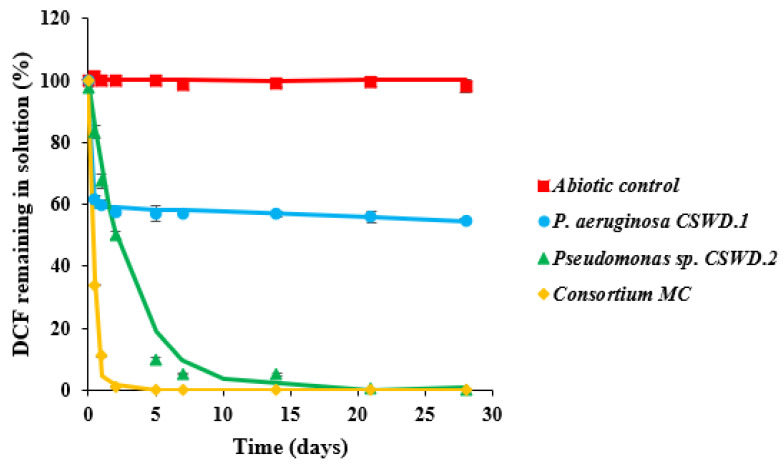
Biodegradation curves of 10 mg L^−1^ of diclofenac (DCF) in solution under different treatments. Abiotic control (■), CSWD.1 strain (●), CSWD.2 strain (▲), and consortium MC (♦). Solid lines show model fitting to the experimental results (symbols). Error bars indicate standard deviation from the replicates.

This study presents the biodegradation performance of the two bacterial strains previously identified as *P. aeruginosa* CSWD.1 and *Pseudomonas* sp. CSWD.2, as well as the consortium “MC”, all of which demonstrated notable degradation capacity. Based on the results obtained from the enrichment culture (Section 3.1), co-metabolism with glucose was found to enhance DCF degradation significantly. Therefore, in our study, a glucose concentration of 3 g L^−1^ was supplemented to the 10 mg L^−1^ DCF solution. The concentration used for the biodegradation studies in the present work is higher than those found in WWTPs and other environmental matrices, to ensure measurable biodegradation and assess the capabilities of microorganisms under controlled conditions. Other authors followed similar approaches [28,29,33,44]. The degradation curves obtained are shown in Figure 3, and the corresponding kinetic parameters are given in Table 3. 

**Figure 4:**
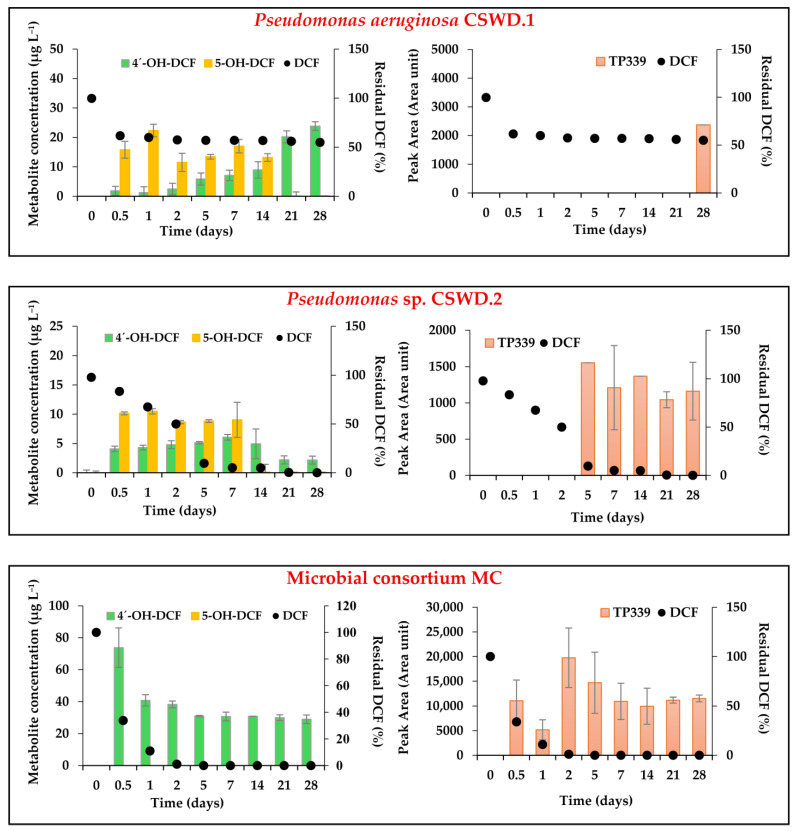
DCF metabolites and transformation products in solution during the biodegradation of DCF in the presence of strains CSWD.1 and CSWD.2, and consortium MC. Vertical bars represent standard deviations.

In addition to monitoring DCF concentration, some metabolites and transformation products of DCF were also analyzed: 4’-OH-DCF, 5-OH-DCF, 1-O-acylglucuronide diclofenac, 4,5-dihydroxydiclofenac, TP339 (nitration), TP323 (nitrosation), and 5-hydroxydiclofenac lactam, detecting the presence of only 4’-OH-DCF, 5-OH-DCF, and TP339 in our samples (Figure 4).

When the DCF biodegradation assay was conducted using strain CSWD.1, a significant degree of biotransformation was observed in the first 24 h, with 40% degraded, although the degradation slowed considerably from this point on. The curve of residual DCF concentration fitted a biphasic first-order sequential model (HS). The model-based calculations indicated approximately up to 45.5% degradation after 28 days and a DT_50_ value of around 57.5 days. During the initial stage of the assay, DCF biodegradation occurred rapidly, with a rate constant (K_1_) of 1.05 day^−1^. After this period, the degradation rate slowed down, yielding a second-rate constant (K_2_) of 0.002 day^−1^. The slowdown observed in the biodegradation process could be attributed to the damage caused by DCF itself and by its metabolites and/or transformation products to the microorganisms responsible for degradation. Studies have demonstrated that DCF can induce changes in microbial growth by compromising cell membrane integrity, modifying enzymatic functions, and triggering oxidative stress through the production of reactive oxygen species (ROS), which in turn can damage essential cellular constituents such as lipids, proteins, and nucleic acids [18]. Ivshina et al. [29] observed morphological changes and impaired growth in *Rhodococcus ruber* IEGM 346 when exposed to high concentrations of DCF (50 mg L^−1^). In another study, exposure of *Pseudomonas moreii* KB4 to DCF (1–3 mg L^−1^) affected cellular physiology by decreasing the zeta potential and increasing the cell wall hydrophobicity [27]. In addition, Matejczyk et al. [67] observed a reduction in the viability test of *E. coli* in the presence of 4’-OH-DCF and 5-OH-DCF, the main DCF metabolites.

In the presence of *P. aeruginosa* CSWD.1, the analysis of metabolites formed during the biodegradation process revealed a continuous increase in the concentration of 4’-OH-DCF throughout the 28-day incubation period (Figure 4). This compound, identified as the main human metabolite of DCF and also detected in wastewater [81], has been described as one of the major bottlenecks in DCF degradation. Although 4’-OH-DCF is transformed more rapidly than the parent compound, its formation from DCF through hydroxylation constitutes the rate-limiting step in the overall degradation pathway [13,26]. Conversely, 5-OH-DCF was detected within the first 12 h at concentrations higher (16 µg L^−1^) than those of 4’-OH-DCF (2 µg L^−1^). This trend persisted until day 14, after which 4’-OH-DCF became predominant (24 µg L^−1^ after 28 days), indicating that CYP450 monooxygenase played a key role in the initial step of DCF degradation, as it is responsible for the hydroxylation of DCF to form 4′-OH-DCF and 5-OH-DCF [82]. This pattern may be explained by the fact that, initially, hydroxylation of the aromatic ring generates both metabolites, but 5-OH-DCF displays greater chemical reactivity [83]. As a result, it is more susceptible to further oxidation reactions, such as the formation of benzoquinone imine, a highly reactive intermediate that rapidly undergoes additional oxidation or conjugation, thereby reducing its concentration in the system. In contrast, 4’-OH-DCF is a more stable metabolite and can accumulate during the intermediate phase of biodegradation, as its transformation into final products proceeds more slowly [65]. In addition, only after 28 days was TP339, a nitrated transformation product (Table S3), detected (Figure 4). According to current literature, information regarding the nitroso and nitro derivatives of DCF remains scarce. The first evidence of these metabolites in WWTPs was reported by Osorio et al. [68], who demonstrated that DCF can undergo biotransformation into nitroso and nitro derivatives under biological conditions in laboratory-scale bioreactors inoculated with WWTP mixed liquor. Likewise, Chiron et al. [84] observed the formation of nitrogen-containing derivatives, N-nitroso-DCF (NO-DCF) and 5-nitro-DCF (NO_2_-DCF), in soil under anoxic conditions. More recently, Osorio et al. [85] identified uncommon microbial nitration and nitrosation TPs of DCF-related compounds in bioreactors containing WWTP mixed liquor. These findings are consistent with our results, and TP339 was tentatively identified as NO_2_-DCF based on MRM transitions, with identification supported by characteristic fragment ions and retention time matching, consistent with previously reported data (Table S3).

In the case of *Pseudomonas* sp. CSWD.2, the biodegradation results were highly significant; the biodegradation curve fitted a single first-order kinetic model (SFO), with 50% DCF removal achieved in just 2 days, 90% in 5 days, and complete removal after 21 days (Figure 3). The formation of the metabolites 4’-OH-DCF and 5-OH-DCF fluctuated throughout the biodegradation process. Specifically, 5-OH-DCF disappeared after 7 days of incubation, while 4’-OH-DCF persisted, reaching a concentration of 2 µg L^−1^ at the end of the experiment (Figure 4). In contrast, TP339 was detected from day 5 onward (1554 peak area units) and remained detectable at the end of the 28-day treatment period (1162 peak area units).

The ability of individual bacterial strains to degrade DCF has been previously demonstrated; however, fewer than twenty isolates have been reported as DCF degraders over the past decade [18]. Notable examples of bacterial strains that use DCF as a sole carbon and energy source were summarized in Section 1. Following an exhaustive review, Badawy et al. [18] concluded that, from a phylogenetic diversity perspective, fourteen bacterial genera have been identified as DCF degraders, among which *Pseudomonas*, *Klebsiella*, and *Enterobacter* accounted for the largest proportions of isolates (12.5% each). On the other hand, studies indicate that microbial consortia frequently outperform single bacterial isolates in degrading complex pollutants, including pharmaceuticals, due to their metabolic synergy, enzymatic complementarity, and greater stability under variable environmental conditions [86,87,88]. Cooperative metabolic interactions, such as cross-feeding and co-metabolism, enable consortia to degrade contaminants that individual strains cannot remove, while also enhancing their adaptability and efficiency in the simultaneous removal of multiple compounds [25,89]. Moreover, since bacteria grow more rapidly and fungi produce more potent enzymes, fungal-bacterial consortia tend to outperform monocultures in their ability to degrade resistant contaminants [90].

In our study, the experimental biodegradation data obtained for the consortium isolated from the glucose-enriched culture (MC) were fitted to the SFO kinetic models described by FOCUS [48]. A 66% reduction in the initial DCF concentration (10 mg L^−1^) was observed after 12 h of incubation (Figure 3), and complete biodegradation was achieved within 5 days, with a DT_50_ value of only 0.3 days (Table 3). In contrast to isolates, the metabolite 5-OH-DCF has not been detected during the biodegradation process (Figure 4). The formation of the metabolite 4’-OH-DCF fluctuated throughout the biodegradation process (Figure 4). After 0.5 days, its concentration was the highest (73.8 µg L^−1^) and began to decrease, but remained at a concentration of 29 µg L^−1^ after 28 days. On the other hand, TP339 was detected from day 0.5 (11,072 peak area units) and remained detectable at the end of the 28-day treatment period (11,531 peak area units). It is worth highlighting that the TP339 peak area is much higher than in the presence of isolated bacteria.

Focusing on studies addressing DCF degradation through microbial consortia, a bacterial consortium composed of *Enterobacter hormaechei* D15 and *Enterobacter cloacae* D16 achieved 98% removal of DCF (initial concentration 10 mg L^−1^) and produced metabolites such as 4’-OH-DCF and nitro derivatives, with low ecotoxicity of the final products [37]. Similarly, a consortium comprising *Bacillus thuringiensis* B1 and *Pseudomonas moorei* KB4 demonstrated high efficiency in DCF degradation in sequential reactors, showing resistance to toxic compounds commonly found in wastewater and the ability to operate across a wide range of pH and temperature conditions [38]. In another study, artificial consortia composed of native bacterial and fungal strains from activated sludge achieved over 80% DCF degradation (30 mg L^−1^), mainly through hydroxylation reactions, while also reducing the ecotoxicity of the treated solutions [87]. Murshid and Dhakshinamoorthy [36] achieved 89% biodegradation of DCF at 150 mg L^−1^ within 120 h using a consortium composed of *Alcaligenes faecalis*, *Staphylococcus aureus*, *Staphylococcus haemolyticus*, and *Proteus mirabilis*.

There was no evidence of abiotic DCF dissipation in solution in the abiotic control (Figure 3), indicating that its degradation was exclusively driven by microbial activity. This finding aligns with previous reports highlighting the key role of microorganisms in the breakdown of emerging pollutants. In this context, microbial biodegradation is widely recognized as an effective, sustainable, eco-friendly, and cost-efficient strategy for their removal [45,91]. Some studies have demonstrated the chemical mineralization of DCF [92,93]. However, according to Wojcieszyńska et al. [1], only a few bacterial isolates have been able to achieve complete mineralization of DCF, due to the challenges associated with degrading the intermediate transformation products formed during its biodegradation. For instance, Mohamed et al. [82] proposed degradation pathways for *Pseudomonas aeruginosa* S1, *Achromobacter* sp. S11, and *Achromobacter piechaudii* S18, demonstrating the formation of metabolites following aromatic ring cleavage and CO_2_ production, which is indicative of mineralization. In another study, exposure of *Klebsiella* sp. KSC to 70 mg L^−1^ of DCF resulted in the mineralization of DCF after 72 h, despite the continued presence of a wide range of metabolites [33].

The presence of such intermediates in the solutions after the DCF degradation process is extensively demonstrated. For this reason, ecotoxicity bioassays were performed on the post-biodegradation solutions to verify if the presence of biotransformation products or metabolites generated during the process could generate higher toxicity than the parent contaminant.

### 3.4. Ecotoxicity Studies in Diclofenac-Contaminated Aqueous Samples

The non-inoculated DCF solutions (10 mg L^−1^) and the supernatant samples collected at the end of the biodegradation process (after 28 days) in the presence of CSWD.1, CSWD.2 strains, and consortium MC were used to perform ecotoxicity assays in triplicate (Table 4), and their relation to the presence of DCF metabolites produced throughout the biodegradation process was evaluated (Figure 4).

The initial DCF concentration was categorized as presenting acute toxicity (1 < TU < 10) according to the criteria established by Persoone et al. [52], with a calculated TU value of 6.6 and an EC_50_ of 15.2%. Similar toxicity levels for DCF were reported by Aissaoui et al. [37], who used the same initial concentration (10 mg L^−1^) and obtained an EC_50_ of 13.8%. In other studies, Ferrari et al. [94] reported an EC_50_ of 11.45 mg L^−1^. These results confirm that *A. fischeri* is highly sensitive to DCF, supporting the notion that this pharmaceutical compound poses a toxic threat when released into aquatic environments.

When bioaugmentation was performed using the CSWD.1, CSWD.2 strains, and MC, an increase in toxicity was detected, as reflected by higher TU values (Table 4). After 28 days of incubation, the samples exhibited high acute toxicity (TU values of 20.9, 10.5, and 14.3, and EC_50_ of 4.8%, 9.5%, and 7.0% for CSWD.1, CSWD.2 strains, and MC, respectively), suggesting the formation of intermediate metabolites with toxic properties during the biodegradation process. The degradation of DCF involves several key biochemical reactions, including hydroxylation, dehydroxylation, decarboxylation, and dechlorination. These processes lead to the formation of major metabolites such as 4’-OH-DCF, 5-OH-DCF, 3′-hydroxydiclofenac, 4,5-dihydroxydiclofenac, and quinone imine [28,34].

In our study, the analytical determination of several metabolites was carried out, including 4’-OH-DCF and 5-OH-DCF, and a tentative identification of 1-O-acylglucuronide diclofenac, 4,5-dihydroxydiclofenac, 5-hydroxydiclofenac lactam, TP339 (nitration transformation product), and TP323 (nitrosation transformation product) was performed. However, only 4’-OH-DCF, 5-OH-DCF, and TP339 (nitration transformation product) were detected in the samples as previously commented. In the case of *P. aeruginosa* CSWD.1, 4’-OH-DCF exhibited its maximum concentration (24 µg L^−1^) at the end of the biodegradation process (Figure 4), and also in the case of *Pseudomonas* sp. CSWD.2, 4’-OH-DCF continued to be present at the end of biodegradation (2 µg L^−1^ after 28 days). Regarding metabolite analysis of MC, the metabolite 5-OH-DCF was not detected, but 4’-OH-DCF was detected throughout the entire biodegradation process, remaining after 28 days of incubation (29 µg L^−1^) (Figure 4). Summarizing, at the end of the biodegradation process (28 days), 4’-OH-DCF was detected in all cultures inoculated with the different microorganisms. The results obtained here suggest that the degradation process under our experimental conditions was dominated by oxidative reactions, leading to the formation of hydroxylated compounds, such as 4’-OH-DCF. These oxidative pathways could account for the increase in toxicity observed in the bioassays [26]. Matejczyk et al. [67] reported a toxic synergistic effect between DCF and its hydroxylated metabolites, supporting the hypothesis that these intermediate compounds may be more harmful than DCF itself.

Several studies have pointed out the toxicity of DCF using the Microtox system [37,94,95,96]. However, few studies have been conducted that evaluated the toxicity of DCF together with its metabolites. Pápai et al. [32] demonstrated that *Stenotrophomonas humi* strain DIC_5 was able to produce metabolites that increased toxicity compared with the initial value during the DCF biodegradation process, although the specific metabolites responsible were not identified. Grandclément et al. [97] reported that the observed ecotoxicity after 30 min of DCF (EC_50_ = 23 mg L^−1^) was lower than 4’-OH-DCF (EC_50_ = 19 mg L^−1^). In another study, toxicity assessments of DCF and its metabolites were conducted using acute and chronic toxicity data for fish (96 h), *Daphnia* (48 h), and green algae (96 h). The results showed that the ecotoxicity of the parent compound DCF was generally equivalent to or higher than that of its identified metabolites, except for 4’-OH-DCF and 5-OH-DCF, which exhibited slightly higher toxicity [98].

In addition, a nitro derivative of DCF (NO_2_-DCF/TP339) was identified in the biodegradation samples, but without an absolute concentration. In the case of CSWD.1, it was detected only after 28 days (2374 area units). However, for CSWD.2 strain TP339 appears at 5 days, and the peak area fluctuates along with the biodegradation, continuing at 28 days (1162 area units). In the MC, its signal began to increase after 0.5 days of incubation (11,072 area units), maintaining a similar peak area after 28 days (11,531 area units), which is approximately tenfold higher than in the case of CSWD.1 and CSWD.2 strains. Information has been reported in the literature about the toxicity of nitrated transformation products of DCF is scarce. Osorio et al. [68] reported that NO-DCF did not exhibit acute toxicity in Microtox assays using *V. fischeri*, both in wastewater and receiving surface waters; the EC_50_ value for DCF was 22.9 mg L^−1^, whereas NO-DCF displayed higher EC_50_ values (>100 mg L^−1^), indicating lower toxicity than its parent compound. In contrast, NO_2_-DCF was found to be slightly more toxic to *V. fischeri* (EC_50_ = 11.7 mg L^−1^).

Based on the previously reviewed literature, it can be concluded that in the case of CSWD.2, the bacterial strain that achieved the lowest TU values (10.5), the observed toxicity could be attributed to the tentative presence of the metabolites 4’-OH-DCF and NO_2_-DCF, which were detected at lower signals than in CSWD.1 and MC. The MC treatment showed an intermediate TU value (14.3), likely because the concentrations of 4’-OH-DCF and the peak area of NO_2_-DCF were higher than those obtained with CSWD.2 inoculation. Finally, the application of CSWD.1 resulted in the highest TU value (20.9), not only due to the presence of 4’-OH-DCF and NO_2_-DCF but also because of the remaining DCF (55% of the initial concentration), which persisted in solution at 5.5 mg L^−1^.

Overall, these findings indicate that although highly efficient DCF-degrading strains are available, it is essential to assess the metabolites produced and their associated toxicity to accurately determine the effectiveness of the decontamination strategy. Therefore, it is crucial to continue this work by identifying other microorganisms capable of degrading the detected compounds and using them in combination with the strains described in this study.

### 3.5. Shift in Bacterial and Fungal Communities in the Consortium Based on DNA Metabarcoding Data

**Figure 5:**
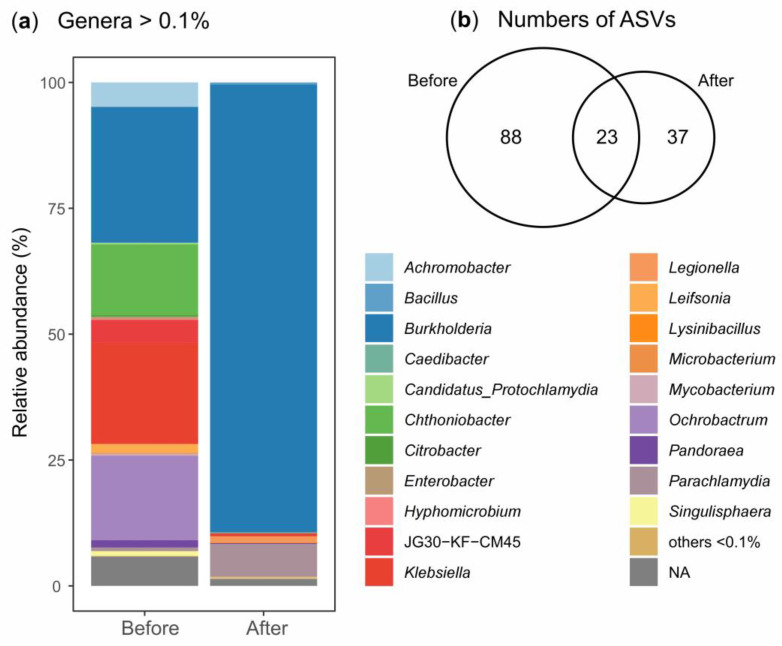
(**a**). The most abundant (>0.1%) bacterial genera identified in the study samples (before and after). Relative abundance data are percentages of rarefied sequences per sample. ASVs without identification at the genus level were collapsed in the category NA. (**b**). Venn diagram showing the overlap between samples, with 23 of 148 (15%) ASVs present in both samples.

DNA metabarcoding data (16S and ITS2) enabled us to compare the microbial communities (bacteria and fungi) in two MC samples collected at the beginning and the end of the DCF degradation experiment. After quality filtering and rarefaction, the complete lists of bacterial (148) and fungal (32) ASVs detected in this study, detailing their abundance, distribution, taxonomic assignments, and representative DNA sequence, are available as Supplementary Tables S5 and S6, respectively. Bacterial species richness (observed ASVs) decreased from 111 to 60 during the degradation experiment (Table S4 and Figure 5b). This reduction may result from stress induced by organic contaminants, which can damage microorganisms and limit their overall growth [99]. Moreover, contaminants can exert a direct stimulatory effect on microbial populations capable of tolerating and/or degrading them [100]. In the specific case of DCF, previous studies have shown that exposure to this compound can induce substantial shifts in the structure and composition of bacterial communities, promoting the proliferation of genera capable of degrading the drug while reducing overall diversity, particularly under enrichment conditions where DCF serves as the sole carbon source [69,101]. During the enrichment process, a marked increase in the relative abundance of certain genera is often observed, whereas other taxa decline, reflecting possible shifts in community composition and function. This phenomenon aligns with the selective pressure exerted by DCF, which favors microorganisms possessing specialized metabolic pathways for degrading aromatic and xenobiotic compounds [102].

**Figure 6:**
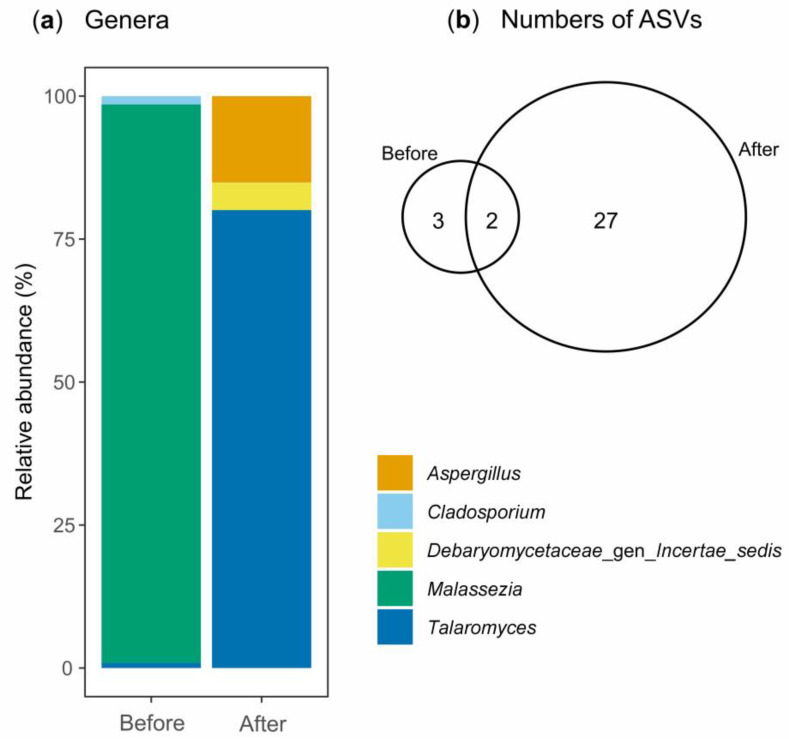
(**a**). Fungal genera identified in the study samples (before and after). Relative abundance data are percentages of rarefied sequences per sample. ASVs without identification at the genus level were collapsed in the category NA. (**b**). Venn diagram showing the overlap between samples, with 2 of 32 (6.25%) ASVs present in both samples.

However, in contrast to bacterial richness, fungal richness increased from 5 to 29 after DCF degradation processes (Table S4 and Figure 6b). This observation can be interpreted as an adaptive response of the initial rare fungal community (a few ASVs with low numbers of sequences) to the presence of the drug and its metabolites. During the biodegradation process, the selective pressure exerted by DCF may promote the proliferation of fungal species that were initially rare or undetectable due to their capability of metabolizing the compound or resisting its toxic effects [103,104]. The literature indicates that fungi possess a remarkable ability to tolerate and transform DCF, owing to their metabolic diversity and the production of oxidative enzymes such as laccases and peroxidases, which facilitate the degradation of NSAIDs, including DCF [105,106].

Bacterial and fungal communities are shown at genus levels in Figure 5a and Figure 6a, respectively. Bacterial metabarcoding data revealed a shift in the community composition throughout the DCF biodegradation process. Initially, the bacterial community was dominated by the genera *Burkholderia* (26.9% in relative abundance), *Klebsiella* (20.1%), *Ochrobactrum* (16.8%), and *Chthoniobacter* (14.1%) (Figure 5a), reflecting a structure typically observed in consortia previously exposed to aromatic contaminants and pharmaceuticals. This pattern is consistent with previous enrichment studies involving DCF and other pharmaceuticals. In this regard, Suleiman et al. [41] reported that *Burkholderia* and *Klebsiella* were among the most abundant genera in batch cultures exposed to various pharmaceuticals, highlighting their potential role in microbial adaptation to these compounds.

After 28 days of incubation in the presence of DCF, *Burkholderia* represented 88.9% of the community, suggesting it became highly abundant under the experimental conditions. Other genera also increased in relative abundance, although to a lesser extent, such as *Parachlamydia* (from 0.7% to 6.6%) and *Legionella* (from 0.02% to 1.2%). Conversely, the striking decline of *Klebsiella* (from 20.1% to 0.6%) may be attributed to its typical prevalence in sludge and wastewater environments, where it exhibits tolerance to certain pollutants. However, it could be outcompeted by more specialized taxa under a prolonged exposure to DCF, as demonstrated by Farkas et al. [107].

The shift in the bacterial community was also reflected by the relatively low overlap (15%; 23 of 148 ASVs) between samples (before vs. after; Figure 5b).

The clear dominance of *Burkholderia* suggests that this genus has undergone an increase in relative abundance during the incubation under the selective pressure exerted by DCF as the primary substrate. This phenomenon is consistent with reports indicating that sustained exposure to pharmaceutical compounds promotes the selection of bacterial genera capable of tolerating and metabolizing such xenobiotics, including *Burkholderia*, *Pseudomonas*, *Achromobacter*, and *Cupriavidus* [41,101].

Although no *Burkholderia* strains have yet been identified as direct degraders of DCF, this genus displays remarkable catabolic versatility toward xenobiotic aromatic compounds [108,109]. In a comparative genomic analysis of 80 *Burkholderia* species, Pérez-Pantoja et al. [108] demonstrated that these bacteria possess nearly all central ring-cleavage pathways and most peripheral pathways required for aromatic compound catabolism. Among the most widely distributed ring-cleaving enzymes are protocatechuate 3,4-dioxygenase (*Pca34*), catechol 1,2-dioxygenase (*Cat12*), and homogentisate 1,2-dioxygenase (*Hge*). These three ortho-cleavage pathways, protocatechuate, catechol, and homogentisate, are among the most prevalent, being detected in at least 60% of the genomes analyzed, along with the phenylacetyl-CoA (*Paa*) degradation pathway [108].

The degradative potential of *Burkholderia* has been demonstrated for a variety of aromatic compounds, including toluene [110], benzene [111], pentachlorophenol [112], and polycyclic aromatic hydrocarbons [109]. However, no direct evidence currently supports its involvement in DCF biodegradation. Nevertheless, its broad metabolic flexibility suggests that *Burkholderia* could participate, directly or synergistically, in DCF degradation processes.

Previous studies on DCF biodegradation have identified other bacterial genera capable of metabolizing this compound, including *Labrys*, *Enterobacter*, *Stenotrophomonas*, *Rhizobium*, *Pseudomonas*, and *Sphingopyxis* [30,37,69,101]. *Burkholderia* has been described as a degrader of other pharmaceuticals, although studies on its pharmacodegradative potential remain limited. For example, Reis et al. [113] reported the presence of a penicillin utilization operon (*put*) in *Burkholderia* strains, which enables the breakdown of penicillin G via β-lactamases and amidases, yielding benzylpenicilloic acid and phenylacetic acid. Similarly, Takenaka et al. [114] demonstrated that *Burkholderia* sp. strain AK-5 utilizes 4-aminophenol, the main metabolite of paracetamol, as the sole source of carbon, nitrogen, and energy. More recently, Wu et al. [115] addressed the issue of carbamazepine pollution, achieving a removal rate of 73.56% within 72 h through microbial communities that included the genus *Burkholderia*.

The rare fungal community before exposure to DCF was represented by the genera *Malassezia* (97.6%), *Cladosporium* (1.4%), and *Talaromyces* (0.9%). The main ecological niche of *Malassezia* is the human skin, where it utilizes lipids as a source of carbon and energy [116]. Recent studies have nevertheless reported its occurrence in other environments, including aquatic systems, deep-sea sediments, Antarctic soils, and corals [117,118]. In these contexts, its abundance is often linked to human-derived contamination. The dominance of *Malassezia* in this microbial consortium from wastewater sludge may thus reflect the input of human biological material. Since this genus is primarily associated with hospitals [119] and the human skin microbiota [116], its transfer to WWTPs is plausible.

Regarding *Cladosporium* and *Talaromyces*, which were minor constituents of the initial community, both are among the most frequently detected fungi in WWTPs, together with *Aspergillus*, *Fusarium*, *Penicillium*, *Candida*, *Trichoderma*, *Saccharomyces*, and *Acremonium* [120]. The genus *Talaromyces* dominated the community after complete DCF degradation (80.1%), followed by *Aspergillus* (15.1%) and a member of the *Debaryomycetaceae* family (4.8%). Its abundance at the end of the experiment may reflect the possible involvement of this genus in DCF transformation.

Similar to bacteria, the fungal community showed a pronounced shift with a low overlap (6.2%; only 2 of 32 ASVs) between samples (before vs. after; Figure 6b). In agreement with the results obtained in this study, the genus *Talaromyces* has been previously reported as a DCF degrader in a single study. Conejo-Saucedo et al. [121] observed that, along with *Mycosphaerella*, *Cuniculitrema*, and *Filobasidium*, *Talaromyces* was one of the most abundant genera in various samples of sewage sludge. Additionally, three *Talaromyces* species were isolated and evaluated in DCF biodegradation assays in solution at an initial concentration of 32 mg L^−1^, where only two were able to consume DCF. *Talaromyces gossypii* consumed approximately 85% and *Talaromyces verruculosus* 37% of the initial concentration after 70 h.

In the case of *Aspergillus*, the second most abundant genus in the MC at the end of the degradation process, this genus has been widely reported as an efficient degrader of DCF. For instance, *Aspergillus niger* degraded 37% of an initial DCF concentration of 2000 mg L^−1^ after 60 days of incubation [122]. The same species achieved a 98% DCF removal after 14 days at a lower initial concentration (1 mg L^−1^) [123]. Similarly, *A. niger* degraded 74% of an initial concentration of 5 mg L^−1^ under experimental conditions of pH 3 and the presence of NaCl [124]. Aracagök et al. [125] also reported complete degradation of 50 mg L^−1^ of DCF by *A. niger* after 48 h of incubation. Other *Aspergillus* species have also demonstrated substantial DCF removal capacity. For example, *A. luchuensis* completely (>99.9%) removed DCF from a synthetic wastewater medium within 10 days of incubation [126]. Likewise, *A. tabacinus*, *A. terreus*, and *A. cejpii* achieved 76%, 49.7%, and 4.6% degradation, respectively, of an initial DCF concentration of 32 mg L^−1^ after 72 h [121].

The family *Debaryomycetaceae*, the third most abundant group in our study, comprises several genera of ascomycetous yeasts. According to phylogenetic and taxonomic analyses, the genera recognized within this family include *Debaryomyces*, *Meyerozyma*, *Lodderomyces*, *Spathaspora*, and *Hemisphaericaspora* [127]. To date, there is no scientific evidence directly linking these genera to the biodegradation of DCF. However, some members of the family have demonstrated the ability to degrade other persistent organic pollutants. For instance, *Debaryomyces hansenii* removed approximately 70% of an initial concentration of 100 mg L^−1^ of benzo(a)pyrene, a highly toxic and mutagenic polycyclic aromatic hydrocarbon, after 3 days of incubation [128]. Similarly, *Meyerozyma caribbica* exhibited a biodegradation efficiency of 77.5% toward lindane (hexachlorocyclohexane) at a concentration of 750 mg L^−1^ within 10 days [129].

Overall, the DNA metabarcoding analysis revealed a clear shift in specific bacterial and fungal taxa in response to DCF exposure. While bacterial richness decreased, the dominance of genera such as *Burkholderia* suggests potential functional roles in the transformation of aromatic xenobiotics. Conversely, the fungal community showed increased richness, with *Talaromyces*, *Aspergillus*, and members of the *Debaryomycetaceae* family emerging as the most abundant taxa at the end of the experiment. These results suggest that fungi could be involved in the degradation of DCF. Overall, the findings point to a potential interaction between bacterial and fungal populations under xenobiotic stress.

## 4. Conclusions

From the enrichment cultures carried out with activated sludge in the presence of DCF, two bacterial strains, *P. aeruginosa* CSWD.1 and *Pseudomonas* sp. CSWD.2, and a microbial consortium (MC) able to degrade DCF were obtained. Strain CSWD.1 achieved a biodegradation extent of 45.5% after 28 days; however, as it is an opportunistic pathogen, its use is restricted to laboratory studies and requires biosafety-aware considerations, precluding its direct environmental application. In contrast, strain CSWD.2 and the MC exhibited much higher degradation potential, achieving complete removal of an initial concentration of 10 mg L^−1^ DCF after 21 days and 5 days, respectively. Metabolite analysis revealed the formation of 4’-OH-DCF, 5-OH-DCF, and NO_2_-DCF during the biodegradation process. To assess the feasibility of the biodegradation strategy, ecotoxicity was evaluated using the Microtox test at the beginning and at the end of the bioremediation process. The initial DCF concentration exhibited acute toxicity (TU = 6.6). At the end of the degradation process in the presence of the different DCF-degrading microorganisms used, 4’-OH-DCF and putative NO_2_-DCF were present, and toxicity increased, reaching high acute toxicity levels (TU = 20.9 for CSWD.1, TU = 10.5 for CSWD.2, and TU = 14.3 for MC). These results highlight the necessity of integrating toxicological assessments with pollutant removal studies. The observed toxicity was attributed to both identified (4’-OH-DCF and putative NO_2_-DCF) and possible unidentified metabolites, emphasizing the importance of evaluating metabolite formation and toxicity. Consequently, isolating additional microorganisms capable of degrading 4’-OH-DCF and NO_2_-DCF is crucial to enhance bioremediation when used in combination with the strains described in this study.

The microbial community structure of the MC shifted markedly following DCF degradation. The relative abundance of the genus *Burkholderia* increased substantially, suggesting its potential involvement in DCF transformation. *Parachlamydia* and *Legionella* also increased, though to a lesser extent. Fungal diversity also expanded after complete DCF biodegradation, with *Talaromyces* emerging as the dominant genus, followed by *Aspergillus* and a member of the *Debaryomycetaceae*. These results suggest that both bacterial and fungal members of the consortium may be involved in DCF degradation.

## Figures and Tables

**Table 1:** Phylogenetic affiliations of bacteria isolated from activated sludge based on their 16S rRNA gene sequences.

Strain (Accession Number)	Phylogenetic Affiliation/Closest Related Sequences (Accession Number)	Similarity (%)	Phylum/Class, Family, Genus
CSWD.1 (PX459929)	*Pseudomonas aeruginosa* (NR_113599.1)	100	*Gammaproteobacteria*/*Pseudomonadales*, *Pseudomonadaceae*, *Pseudomonas*
CSWD.2 (PX660167)	*Pseudomonas* sp. (PX40994.1)	99.92	*Gammaproteobacteria*/*Pseudomonadales*, *Pseudomonadaceae*, *Pseudomonas*

**Table 2:** Inhibitory concentrations (IC_50_) of DCF for specific degrading bacteria and the consortium.

Microorganisms	IC_50_ (mg L^−1^)	R^2^
*Pseudomonas aeruginosa* CSWD.1	7600	0.922
*Pseudomonas* sp. CSWD.2	1100	0.938
Consortium (MC)	25,118	0.943

**Table 3:** Kinetic parameters calculated from DCF dissipation curves in solution under different conditions.

Treatment	Model Kinetic	k_1_ * (Days^−1^)	k_2_ * (Days^−1^)	DT_50_ ** (Days)	Extent of Dissipation *** (%)	Calculated χ^2^ ****	Scaled Error	R^2^
*P. aeruginosa* CSWD.1	HS	1.05	0.002	57.5	45.5	0.2	0.5	0.992
*Pseudomonas* sp. CSWD.2	SFO	0.33	-	2.3	100	10.9	3.9	0.984
*Consortium MC*	SFO	1.75	-	0.3	100	8.5	1.8	0.995

* Degradation rate constants. ** Time to decline to half the initial concentration of DCF. *** After 28 days. **** χ^2^ calculated values < χ^2^ corresponding tabulated value (*p* < 0.05).

**Table 4:** Acute toxicity test towards *V. fischeri* in solution polluted with 10 mg L^−1^ of DCF and 3 g L^−1^ of glucose in the presence of *P. aeruginosa* CSWD.1, *Pseudomonas* sp. CSWD.2, and microbial consortium MC.

Treatment	Time (Days)	TU *	EC_50_ (%) **	Toxicity ***
Abiotic control	0	6.6 ± 0.2	15.2 ± 0.4	Acute toxicity
*P. aeruginosa* CSWD.1	28	20.9 ± 3.5	4.8 ± 1.2	High acute toxicity
*Pseudomonas* sp. CSWD.2	28	10.5 ± 0.3	9.5 ± 0.1	High acute toxicity
Consortium MC	28	14.3 ± 1.7	7.0 ± 0.3	High acute toxicity

* TU: toxic units. ** The EC50 indicates the concentration (% *v*/*v*) required to induce a toxic effect in 50% of *A. fischeri*. *** According to Persoone et al. [52].

## Data Availability

The original data presented in the study are openly available in the European Nucleotide Archive (ENA) under accession number PRJEB98466 (https://www.ebi.ac.uk/ena/browser/view/PRJEB98466, accessed on 1 January 2026).

## Supplementary Materials

The following supporting information can be downloaded at: https://www.mdpi.com/article/10.3390/jox16010024/s1, Figure S1: Growth curve in LB medium of *P. aeruginosa* CSWD.1 and *Pseudomonas* sp. CSWD.2; Table S1: Optimized MRM parameters of the QqQ-MS determination of diclofenac and its metabolites; Table S2: QqQ-MS parameters applied to the identification of diclofenac metabolites/transformation products; Table S3: Structure of studied compounds, extracted from Osorio et al. [50]; Table S4: Metadata of samples analyzed by DNA metabarcoding including their description, accession numbers in the ENA project PRJEB98466, number of reads, as well as the alpha diversity data; Table S5: Complete list of bacterial amplicon sequence variants (148) from this study, detailing their abundance, distribution, taxonomic assignment and representative 16S rRNA gene sequence; Table S6: Complete list of fungal amplicon sequence variants (32) from this study, detailing their abundance, distribution, taxonomic assignment, and representative ITS2 sequence.

## Abbreviations

The following abbreviations are used in this manuscript:

DCF	Diclofenac
MC	Microbial consortium
4’-OH-DCF	4’-Hydroxy-diclofenac
5-OH-DCF	5-Hydroxy-diclofenac
NO_2_-DCF	5-Nitro-diclofenac
NSAIDs	Non-steroidal anti-inflammatory drugs
WWTPs	Wastewater treatment plants
ITS	Internal transcribed spacer
MSM	Mineral salt medium
LB	Luria–Bertani
HPLC	High-performance liquid chromatography
ODS	Octadecylsilane
NCBI	National Center for Biotechnology Information
IC_50_	half-maximal inhibitory concentration
OD	Optical density
LC-MS/MS	Liquid chromatography tandem mass spectrometry
ESI	Electrospray ionization
QqQ	Triple-quadrupole mass
MRM	Multiple reaction monitoring
TP	Transformation product
EC_50_	Concentration (% *v*/*v*) causing toxic effects on 50% of the bacterial population
TU	Toxic units
ENA	European nucleotide archive
ASV	Amplicon sequence variants
SFO	Simple first-order model
HS	Hockey-stick model
BLAST	Basic local alignment search tool
