# Supplementary material for: Enhanced Diclofenac Biodegradation by Bacterial Strains and a Microbial Consortium from Activated Sludge: Toxicity Assessment and Insights into Microbial Community Dynamics
Source: J Xenobiot. 2026 Feb 2;16(1):24. doi: 10.3390/jox16010024 (PMC12921880; doi:10.3390/jox16010024)
Supplement: Supplementary file 1 [file jox-16-00024-s001.zip › jox-4073658-Supplementary Material.pdf]

Journal of *Xenobiotics*

MDPI

Supplementary Materials: Enhanced Diclofenac Biodegradation by Bacterial Strains and a Microbial Consortium from Activated Sludge: Toxicity Assessment and Insights into Microbial Community Dynamics

Alba Lara-Moreno, Belen Rodriguez-Morillo, Fernando Madrid, Pedro M. Martin-Sanchez, Jaime Villaverde, Carmen Mejías, Esteban Alonso, Juan Luis Santos and Esmeralda Morillo

Methodology:

Bacterial growth curves

The pre-inoculum of the selected Diclofenac-degrading bacteria, obtained from an overnight culture, was used to inoculate 100 ml of LB medium in a sterile 250 ml Erlenmeyer flask. Bacterial growth was monitored by measuring the OD_{600} every 2 h until 38 h with a VWR UV-3100 spectrophotometer. The initial OD_{600} of the cultures was set to 0.1, and incubation was with agitation at 125 rpm and 30 ºC.

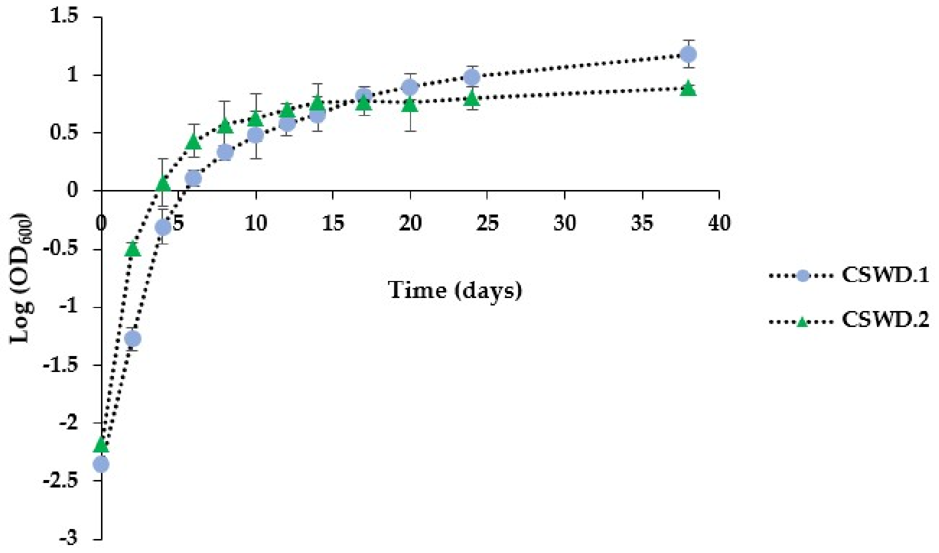

Figure S1. Growth curve in LB medium of *P. aeruginosa* CSWD.1 and *Pseudomonas* sp. CSWD.2.

Table S1. Optimized MRM parameters of the QqQ-MS determination of diclofenac and its metabolites.

Compound	Retention time (min)	Ion polarity	Precursor ion (*m/z*)	MRM1 (quantification)	MRM2 (confirmation)	Ratio	CE (V)
Diclofenac	7.72	Positive	296	296>214	296>250	37.8	32/8
4-Hydroxydiclofenac	6.55	Positive	312	312>230	312>266	46.1	40/8
5-Hydroxydiclofenac	6.71	Positive	312	312>230	312>266	46.1	40/8

CE: collision energy

Table S2. QqQ-MS parameters applied to the identification of diclofenac metabolites/transformation products.

Compound	Ion polarity	Precursor ion (*m*/*z*)	Product ions (*m*/*z*)	CE (V)
1-O-acylglucuronide diclofenac	Negative	470	193,113	12/34
4,5-Dihydroxydiclofenac	Negative	326	282,246	18/26
TP339 (nitration)	Negative	339	295,259	19/32
TP323 (nitrosation)	Positive	295	242,214	27/39
5-Hydroxydiclofenac lactam	Negative	292	228,146	28/28

CE: collision energy; TP: transformation product

Table S3. Structure of studied compounds, extracted from Osorio et al. [50]

Compound	Class	Molecular structure	Molecular formula	Molecular weight
Diclofenac (DCF)	Anti-inflammatory drug	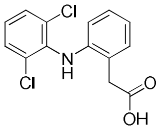	$C_{14}H_{11}Cl_2NO_2$	296.1486
4' -Hydroxydiclofenac (4′-OH-DCF)	Metabolite (hydroxylation)	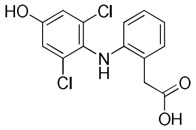	$C_{14}H_{11}Cl_2NO_3$	312.1480
5 -Hydroxydiclofenac (5-OH-DCF)	Metabolite (hydroxylation)	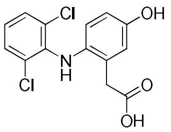	$C_{14}H_{11}Cl_2NO_3$	312.1480
4',5-Dihydroxydiclofenac (4′,5-diOH-DCF)	Metabolite (hydroxylation)	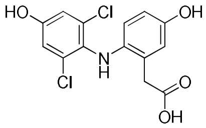	$C_{14}H_{11}Cl_2NO_4$	328.1474
5-OH-DCF-lactam (5-OHD-DCF)	Metabolite (lactam)	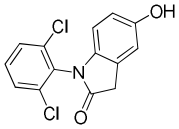	$C_{14}H_9Cl_2NO_2$	294.1328
Diclofenacglucuronide1-β-O-acyl (DCF-gluc)	Metabolite (glucuronidation)	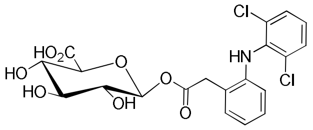	$C_{20}H_{19}Cl_2NO_8$	472.2728
TP339 (NO_2-DCF)	Transformation product (nitration)	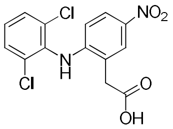	$C_{14}H_{10}Cl_2N_2O_4$	341.1462
TP323 (NO-DCF)	Transformation product (nitrosation)	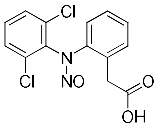	$C_{14}H_{10}Cl_2N_2O_3$	325.1468

Table S4. Metadata of samples analyzed by DNA metabarcoding, including their description, accession numbers in the ENA project PRJEB98466, number of reads, as well as the alpha diversity data.
Table S5. Complete list of bacterial amplicon sequence variants (148) from this study, detailing their abundance, distribution, taxonomic assignment and representative 16S rRNA gene sequence.
Table S6. Complete list of fungal amplicon sequence variants (32) from this study, detailing their abundance, distribution, and taxonomic assignment and representative ITS2 sequence.
